# Chapped Nipples

**Published:** 1842-10-29

**Authors:** 


*Chapped Nipples.*—M. Donné is of opinion that this most troublesome, and often most distressing complaint is, very often at least, connected with an altered state of the milk itself. The milk will, in many such cases, be found to be poor and watery, not very abundant, and to contain more or less of mucous matters. The child, being imperfectly fed, and finding difficulty in drawing the milk, pulls at the nipple more than usual; and perhaps at the same time its saliva is more saline than usual, and thus contributes to increase the irritation.—*Ibid, from Conseils aux Mères, &c.*

